# Supplementary material for: Ascarosides and Symbiotic Bacteria of Entomopathogenic Nematodes Regulate Host Immune Response in Galleria mellonella Larvae
Source: Insects. 2024 Jul 9;15(7):514. doi: 10.3390/insects15070514 (PMC11277396; doi:10.3390/insects15070514)
Supplement: Supplementary file 1 [file insects-15-00514-s001.zip › insects-3034062-supplementary.pdf]

Supplementary Table S1. Primers used in this study

**Primer name**	**Sequence 5´-3´**	**Reference**
Hemolin -F	CTCCCTCACGGAGGACAAAC	Lange et al., 2018
Hemolin -R	GCCACGCACATGTATTCACC	Lange et al., 2018
NOX-F	TGGCACGGCATCAGTTATCA	Lange et al., 2018
NOX-R	ACAGCGACTGTCATGTGGAA	Lange et al., 2018
NOS -F	ATGAAGGTGCTGAAGTCACAA	Lange et al., 2018
NOS -R	GCCATTTTACAATCGCCACAA	Lange et al., 2018
Gallerimycin-F	TATCATTGGCCTTCTTGGCTG	This study
Gallerimycin-R	GCACTCGTAAAATACACATCCGG	This study
Cecropin-F	ATGCTAAGTGCCGTAAGCGG	This study
Cecropin-R	ATGCTTGACCCACAACTGCT	This study
Gloverin-F	GTGTTGAGCCCGTATGGGAA	This study
Gloverin-R	ACGATCGTAGGTGCCTTGTG	This study
GST-F	AGCGAGGGCTCTCAACCTAAATCTT	This study
GST-R	ATTCCCATAGGGAGAATCCATCGTC	This study
16s-F	ACAGAGTTGGATCTTGACGTTACCC	This study
16s-R	AATCTTGTTTGCTCCCCACGCTT	This study
GAPDH-F	GTCATTCCCGCACTTAATGG	This study
GAPDH-R	CAGCTTCCTTGACCTTCTGC	This study

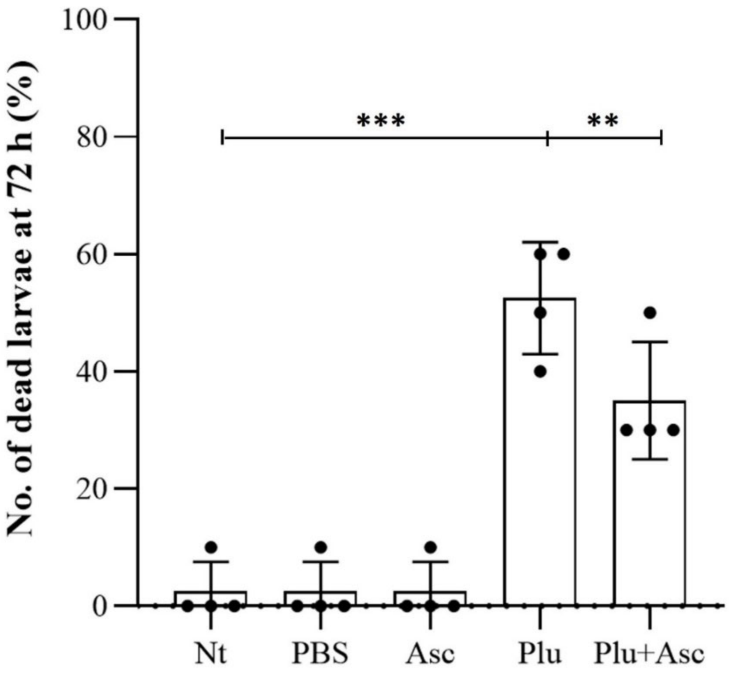

Supplementary Figure S1. The percentages of *Galleria* larvae mortality at 72 h after administration. The abbreviations are referred in Figure 1. The data was analyzed using ANOVA (*P<0.05, **P<0.01, ***P<0.001).
